# Early Life Abuse Moderates the Effects of Intranasal Oxytocin on Symptoms of Premenstrual Dysphoric Disorder: Preliminary Evidence From a Placebo-Controlled Trial

**DOI:** 10.3389/fpsyt.2018.00547

**Published:** 2018-11-29

**Authors:** Erin C. Walsh, Tory A. Eisenlohr-Moul, Cort A. Pedersen, David R. Rubinow, Susan S. Girdler, Gabriel S. Dichter

**Affiliations:** ^1^Department of Psychiatry, University of North Carolina at Chapel Hill School of Medicine, Chapel Hill, NC, United States; ^2^Department of Psychiatry, Neuropsychiatry Institute, University of Illinois at Chicago, Chicago, IL, United States; ^3^Carolina Institute for Developmental Disabilities, University of North Carolina at Chapel Hill School of Medicine, Chapel Hill, NC, United States

**Keywords:** oxytocin, early life abuse, PMDD, emotional symptoms, interpersonal symptoms

## Abstract

**Background:** Although intranasal oxytocin (OXT) has been proposed to be a promising treatment for some psychiatric disorders, little research has addressed individual difference factors that may predict response to OXT. One such factor is early life abuse (ELA), which has widespread influences on social-emotional processing and behavior. This single-blind, placebo-controlled crossover trial examined the role of ELA in shaping the effects of intranasal OXT (vs. placebo) on daily behavioral symptoms in women with three or more prospectively-diagnosed cycling symptoms of premenstrual dysphoric disorder (PMDD).

**Methods:** Participants were ten women with PMDD (*n* = 8) or subthreshold PMDD (*n* = 2), who had experienced ELA prior to age 13 (*n* = 5) or no ELA (*n* = 5). They completed two study visits during the late luteal (premenstrual) phase: once following administration of intranasal OXT and once following intranasal placebo (counterbalanced). Participants then self-administered OXT or placebo at home three times per day for 5 days or until menstrual onset, and prospectively rated daily emotional symptoms of PMDD. Power was adequate to detect medium main and interactive effects.

**Results:** Among women with ELA, intranasal OXT (vs. placebo) increased the premenstrual emotional symptoms of PMDD, whereas among women without ELA, OXT decreased symptoms.

**Conclusion:** This study adds to a growing literature highlighting the importance of considering historical social contexts and traits (such as ELA) as moderators of therapeutic response to OXT.

## Introduction

Premenstrual dysphoric disorder (PMDD) affects 3–8% of reproductive age women (1) and is characterized by the cyclic recurrence of affective, somatic, and interpersonal symptoms during the luteal phase of the menstrual cycle, with full remission of symptoms during the follicular phase (1, 2). Diagnosis with PMDD requires clinically significant changes in one of four core emotional symptoms: anger/irritability, depression, anxiety, and mood swings (1); further, to meet strict diagnostic criteria for PMDD as recently codified in the DSM-5, five or more unique symptoms must demonstrate this cyclical change (3). However, this requirement of five-or-more unique cycling symptoms has been criticized as being too strict, since many women experience fewer cycling symptoms that are nonetheless severe enough to cause cyclical impairment (4, 5). Degree of impairment in women with PMDD or subthreshold PMDD has been found to be equivalent to other mood disorders (1, 5). Interpersonal symptoms, such as anger/irritability and rejection sensitivity, are among the most commonly-observed symptoms in prospectively-diagnosed PMDD (6–8). Treatment with selective serotonin reuptake inhibitors (SSRIs) resolves symptoms in many women with PMDD; however, nearly 40% do not respond (9) signaling the need to develop new treatments and to better match patients to existing treatments. In the present single-blind, randomized, crossover controlled trial of intranasal oxytocin for PMDD, we examined whether early life abuse (ELA) serves as an indicator of premenstrual symptom response to OXT (vs. placebo).

Decades of research point to a role for the neuropeptide oxytocin (OXT) in the regulation of social and emotional behavior. OXT facilitates social bonding and attachment, attenuates stress responses, and reduces anxiety-like behaviors via effects on neural circuitry central to emotional and social processing (10). OXT is synthesized in the paraventricular and supraoptic nuclei of the hypothalamus, and OXT neurons project to brain regions involved in stress regulation (e.g., prefrontal cortex), emotion and salience (e.g., amygdala), and reward (e.g., ventral striatum) (11, 12). Human neuroimaging investigations suggest OXT may improve emotion regulation and interpersonal cooperation via top-down inhibition of arousal and fear responses, and/or enhancement of reward salience, within these neural circuits (12, 13). Accordingly, intranasal OXT has been examined as a treatment in clinical samples with affective and social-cognitive deficits, with several studies reporting superiority to placebo (10, 13).

Although some studies demonstrate a benefit of intranasal OXT on social-emotional behavior and associated brain circuitry, other studies suggest that contextual factors, including history of early life abuse (ELA), heavily moderate these findings (14, 15). Following intranasal OXT administration, participants with a history of ELA show absent or diminished socioemotional benefits (16–19). Furthermore, after OXT administration, null or worsening of effects on social cognition and behavior are observed in individuals with insecure attachment styles (20, 21), which are often associated with ELA (22). These reports are corroborated by neuroimaging investigations documenting differential patterns of OXT-related brain activation or connectivity in individuals with vs. without a history of ELA (23–25). The circuits reported in these cross-sectional studies often overlap with those known to be affected by early adverse experiences and linked to disturbances in social-emotional processing (11). Consequently, ELA-related alterations in social-emotional brain circuits are hypothesized to interact with OXT to enhance the salience of negative social cues, thereby increasing behavioral proclivities for threat, vigilance, and interpersonal conflict (14). Thus, although intranasal OXT may be a promising treatment for some psychiatric disorders—and may be a rational treatment for PMDD—a personalized approach may be necessary since OXT efficacy appears to be dependent on the presence of contextual factors such as ELA.

Notably, PMDD is associated with high rates of ELA relative to controls (26) and we have previously shown that ELA predicts a clinically and pathophysiologically distinct subgroup of women with PMDD characterized by disturbances in stress-related/neuroendocrine systems (27–31). Given the high prevalence of ELA and the centrality of emotional and interpersonal impairment in PMDD (6), PMDD represents an ideal model to investigate the interactive effects of OXT and ELA on social-emotional symptoms.

### Current Study

In a single-blind, placebo-controlled crossover trial in women with PMDD, half of whom had ELA, we investigated the interactive effects of intranasal OXT and ELA on daily ratings of core emotional PMDD symptoms during the symptomatic premenstrual phase. Given previous evidence that ELA diminishes the protective effects of OXT on social and emotional symptoms, we tested the hypothesis that intranasal OXT (compared to placebo) would only improve symptoms during the symptomatic premenstrual phase in women with PMDD (or subthreshold PMDD) without a history of ELA. Consistent with prior reports, we did not expect to observe premenstrual symptom improvement in women with PMDD symptoms with a history of ELA. We suspect that ELA influences behavioral response to intranasal OXT in all women, and we utilize PMDD as an ideal sample to investigate the interactive effects of ELA and OXT on affective and interpersonal symptoms. Hence, we did not include a control group in the present study.

## Methods

### Participants

Recruitment took place in the triangle region of North Carolina. After participating in a PMDD diagnostic feeder study, participants were recruited via e-mail and telephone for a study on the role of oxytocin in PMDD symptoms.

Diagnoses of PMDD (or subthreshold PMDD) were made using a standardized protocol, the Carolina Premenstrual Assessment Scoring System [C-PASS; (2)], to confirm the required cyclical symptom pattern (described in detail below) in daily symptom ratings across two to four cycles. Although the C-PASS scoring system can make the strict DSM-5 diagnosis of PMDD (which requires that 5 or more symptoms, including one emotional symptom, show the required pattern of change across at least two cycles), the C-PASS can also indicate a subthreshold diagnosis for each cycle. This subthreshold diagnosis of PMDD differs from PMDD only in that the premenstrual symptom pattern does not need to occur for 5 or more symptoms; just one core emotional symptom following the pattern is sufficient for the subthreshold PMDD diagnosis. These subthreshold PMDD criteria are equivalent to what has been historically used to make the diagnosis of premenstrual syndrome and PMDD (e.g., 32) prior to the adoption of the strict PMDD criteria in the DSM-5. Finally, since there is no evidence that women given the research diagnosis of subthreshold PMDD differ from women with PMDD with respect to severity, clinical course, or treatment response, this more inclusive threshold was used in the present study. For details on the precise protocol and thresholds used by the C-PASS, we refer the interested reader to the validation paper (2).

The final sample consisted of 10 women (ages: 28–45, *M* = 36.6, *SD* = 5.6) with a prospectively-confirmed diagnosis of PMDD (*n* = 8) or subthreshold PMDD (*n* = 2) and no other serious medical conditions. Full demographics are presented in Table 1. Four of five women in each ELA group met criteria for PMDD, while one in each group met criteria for subthreshold PMDD. The two women diagnosed with subthreshold PMDD each showed the required pattern across 2 months of daily ratings for three symptoms (rather than the five cycling symptoms required for PMDD). Women with ELA reported significantly more severe average premenstrual week symptoms at baseline relative to women without ELA (see Table 1); of note, this mean difference is controlled via the main effect of ELA in the analyses described below. Prior to enrollment, participants underwent medical and psychiatric interviews. Other current psychiatric diagnoses, as measured by the Structured Clinical Interview for DSM-IV-TR disorders (33), were exclusionary. Seven out of 10 participants met criteria for history of a past psychiatric disorder [Major Depressive Disorder, Recurrent Episode: 1; Major Depressive Disorder, Single Episode: 4 (2 with Postpartum Onset); Alcohol Abuse: 2]. Four out of 10 participants were currently prescribed daily medications for PMDD-related symptoms (non-ELA Group: sertraline x 2, ELA Group: venlafaxine, lisdexamfetamine). With regard to psychiatric diagnoses and medications, no differences were observed across ELA groups.

**Table 1:** Sample descriptive information by abuse history.

**Variable**	**Full sample (*N* = 10)**	**PMDD + ELA (*N* = 5)**	**PMDD + No ELA (*N* = 5)**
Age	36.6 (28–45)	35.4 (30–43)	37.8 (28–45)
Race			
White	7 (70%)	4 (80%)	3 (60%)
Black	2 (20%)	1 (20%)	1 (20%)
Central American	1 (10%)	0 (0%)	1 (20%)
Current Psychiatric Medication	4 (40%)	2 (40%)	2 (40%)
Past DSM-IV-TR diagnosis	7 (70%)	4 (80%)	3 (60%)
MDD, recurrent	1 (10%)	0 (0%)	1 (20%)
MDD, Single			
Postpartum onset	2 (20%)	1 (20%)	1 (20%)
No postpartum onset	2 (20%)	1 (20%)	1 (20%)
Alcohol abuse	2 (20%)	2 (40%)	0 (0%)
Mean premenstrual week DRSP total symptom score at baseline	72.28 (26.5)	76.97 (29.5)	67.6 (23.5)

*ELA, Early Life Abuse; MDD, Major Depressive Disorder*.

History of ELA was determined by a validated structured interview (34) commonly used in our previous research and defined as physical or sexual abuse prior to the age of 13. Although some of our prior work has focused on different subtypes of abuse across different age ranges (e.g., sexual abuse before the age of 16), in the present study we did not have hypotheses about specific types of abuse, and therefore used this broad definition of ELA. The of age 13 was used as a cutoff for ELA based on evidence that earlier onset of abuse is linked to greater negative outcomes in adulthood [e.g., (35)]. Physical abuse was coded as present if the participant reported ever experiencing either (1) life threat (i.e., physically attacked with the intent to kill or seriously injure), or (2) other physical abuse (i.e., beaten up, hit, burned). Sexual abuse was coded as present if the participant reported ever experiencing the following forced sexual experiences: (1) a perpetrator touching the participant's breasts, pubic area, vagina, or anus with hands, mouth, or objects; (2) making the participant touch the perpetrator's pubic area or anus with hands, mouth or objects; or (3) vaginal or anal intercourse. Fifty percent (*N* = 5) of our sample reported ELA.

All procedures were approved by the local IRB and participants provided written informed consent. Data collection ended after an unanticipated upgrade at our facility; nevertheless, due to the large number of daily observations in this design, 80% power was achieved to observe conventionally medium sized effects of both OXT and the interaction between OXT and ELA.

### Study Procedures (Premenstrual Phase)

In a single-blind, placebo-controlled crossover design counterbalanced by ELA status, participants completed two laboratory visits, including functional magnetic resonance imaging scans (fMRI not reported in the present study). Each visit occurred during the late luteal (premenstrual) phase, 7–11 days following a positive urine ovulation test. Participants were asked to abstain from nicotine and alcohol for minimally 12 h, and food or drink (except water) for minimally 30 min prior to the visit. During study visits, participants self-administered a placebo or OXT (Syntocinon Spray; 40IU; five inhalations/insufflations per nostril) intranasally with detailed instruction and guidance from study coordinators. Intranasal doses of 24–40 IU of OXT have been administered in clinical trials 2–4 times per day without serious adverse effects and demonstrated potentially clinically meaningful effects (36, 37). Saliva was collected ~30 min following intranasal administration to determine whether the experimental conditions had the expected effects on OXT levels. Immediately following saliva collection, participants completed a 1-h fMRI scan, then were released from the study visit. Following study visits, participants self-administered OXT or placebo three times per day for 5 days or until menstrual onset. Participants were asked to complete a daily log documenting date and time of each intranasal dose. These logs, as well as intranasal vials containing OXT or placebo, were to be returned following each study phase. Participants also completed daily symptom reports of core emotional PMDD symptoms (described below).

### Compliance

Compliance of home intranasal administration was monitored using the daily logs. Participants were marked as “compliant” if they documented completion of all doses. The study team also assessed levels of intranasal vials to determine whether or not the vials were empty or near empty to corroborate log reports.

### PMDD Core Emotional Symptoms and Analyses

Throughout the entire study, participants recorded daily symptoms each evening using the DRSP. Five daily symptom outcomes were selected to examine each of the core emotional PMDD symptoms as described in DSM-5: a depression composite focused on depressed affect (mean of depression, hopelessness, and worthlessness/guilt), an anger/conflict composite (mean of anger/irritability and interpersonal conflict), a rejection sensitivity item, a mood swings item, and an anxiety item. We used composite scores (vs. single items for all domains) to reduce the number of comparisons for this study.

Daily symptoms were predicted in multilevel models in SAS PROC MIXED (METHOD = REML, DDFM = KR), with daily reports nested within women. A REPEATED statement specified an autoregressive (observation−1) structure for within-person error, and a RANDOM statement specified a random intercept and random condition contrast. Daily ratings on insufflation days during the premenstrual phase were predicted from condition (OXT days coded as 1, Placebo days coded as 0; within-person), ELA (ELA = 1, No ELA = 0; between-person), and their interaction. For significant interactions, simple slopes of OXT on daily symptoms were calculated by ELA group.

### Power Considerations

Each woman contributed around 10 days of daily premenstrual symptom ratings from insufflation days (5 days per condition); there were 100 total daily observations. We conducted *post-hoc* power analyses to determine the smallest detectible effect size detectible with 80% power for both the main effect of OXT (vs. placebo) on daily symptoms and the interactive effect of ELA and OXT on daily symptoms. This N adjusted for the average clustering of symptoms within women (i.e., ICC) using the design effect calculation provided by Snijders and Bosker (38). Sensitivity analyses were conducted in G^*^Power to determine the smallest detectible effect size (given 80% power, alpha = 0.05, average symptom intraclass correlation (ICC) = 0.10). For the interaction among ELA and OXT, the smallest detectible effect size was *f* = 0.25. Therefore, despite the small number of women in the study, the repeated measures design and relatively low ICC of daily symptoms allowed for sufficient power to test the hypothesis that OXT would exert conventionally medium effects on daily symptoms, and that OXT and ELA would interact to exert conventionally medium-sized effects on daily symptoms. Of course, generalizability may still be limited by the small sample size.

## Results

### Manipulation Check

#### Salivary OXT

For laboratory visits, salivary OXT significantly increased on OXT (vs. placebo) intranasal administration, suggesting that experimental conditions influenced OXT levels in expected directions. See Appendix for further information.

#### Home Compliance

Two out of 10 participants failed to return daily logs and intranasal vials for both premenstrual study phases; 1 out of 10 participants failed to return daily logs for only one study phase (lost in mail; she reported full completion, supported by diminished vials); and 7 out of 10 participants returned all logs and vials for both study phases. For the returned logs and vials, 100% compliance of intranasal administration was observed based on our definitions. Thus, we have confidence that most participants were compliant with study procedures.

### Main and Moderated Effects of OXT on Daily PMDD Symptoms

**Figure 1:**
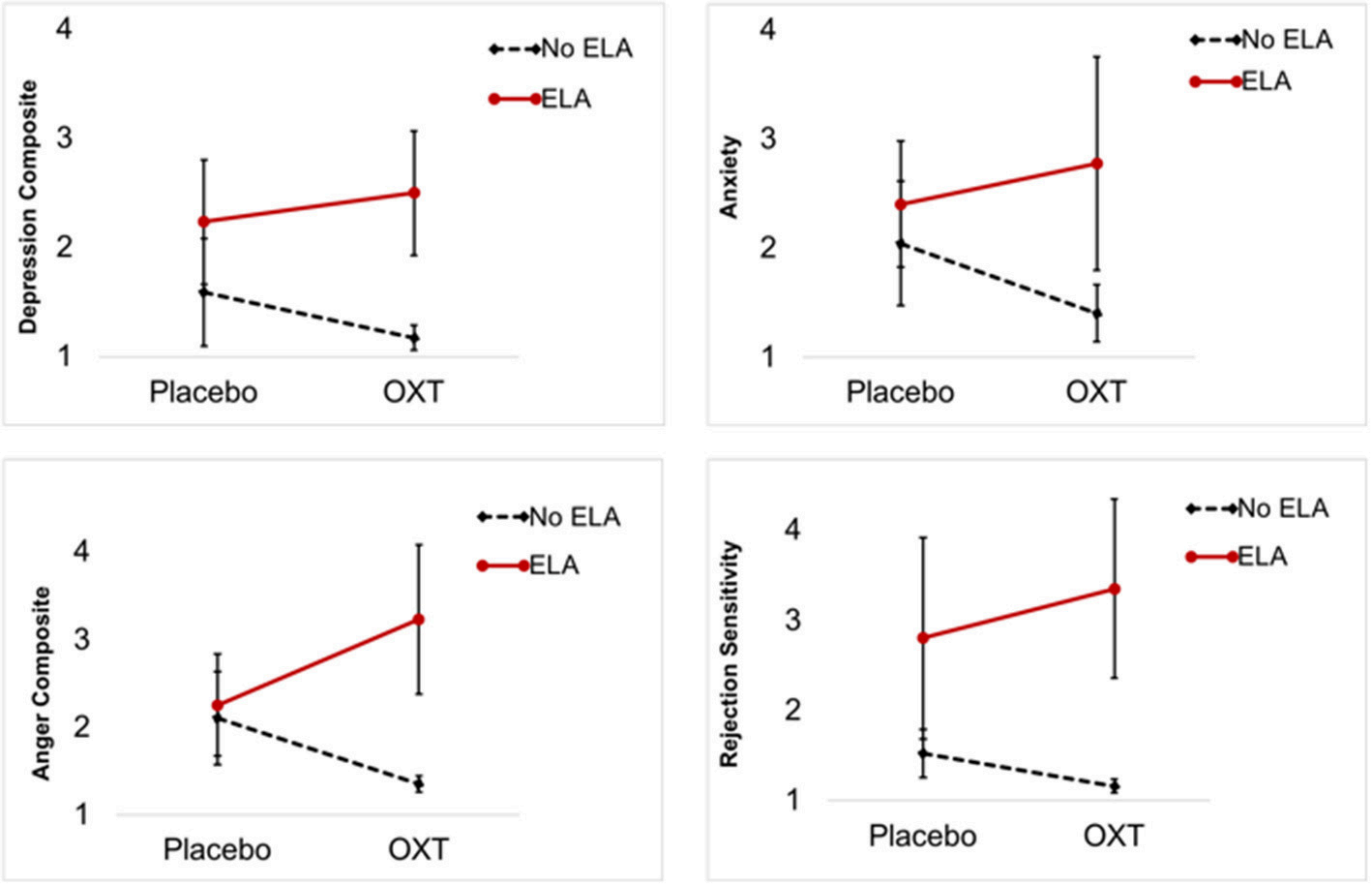
Premenstrual symptom means and standard deviations by ELA group and experimental condition (OXT, Placebo). Each graph illustrates a significant interaction of OXT and ELA on premenstrual symptoms: OXT increased symptoms in PMDD women with ELA and decreased symptoms in PMDD women without ELA.

No significant fixed main effects of OXT on any PMDD symptom were observed (*p's* > 0.05). However, significant random effects of OXT (vs. placebo) indicated a large degree of between-person variability in the effect of OXT on daily symptoms. Thus, the data indicated the presence of individual differences in OXT response. Table 2 presents results of interaction tests. There were significant interactive effects of ELA (between-subject) and OXT (within-subject) on the depression composite, anger/conflict composite, anxiety, and rejection sensitivity—but not mood swings. Simple slopes for significant interactions indicated that OXT (vs. placebo) *decreased* symptoms for women without ELA, but *increased* symptoms for women with ELA (see Table 2 for simple slopes; see Figure 1). Sensitivity analyses were conducted to ensure that our results were not driven by a single participant (e.g., to ensure the results were not caused by the one participant taking lisdexamfetamine, or unique psychiatric histories of various participants); systematic removal of each participant from the model did not lead to substantial changes in the pattern or significance of findings, which reduces concerns about findings being caused by a single multivariate outlier.

**Table 2:** Fixed effects of ELA, oxytocin (vs. placebo), and their interactions on luteal PMDD symptoms on insufflation days.

	**Daily outcomes: core emotional PMDD symptoms**									
**Parameter**	**Depression composite**		**Anger/Conflict composite**		**Anxiety**		**Rejection sensitivity**		**Mood swings**	
	**Estimate**	**(SE)**	**Estimate**	**(SE)**	**Estimate**	**(SE)**	**Estimate**	**(SE)**	**Estimate**	**(SE)**
**FIXED EFFECTS**										
Intercept	1.58	(0.32)	2.10	(0.38)	1.96	(0.46)	1.49	(0.44)	1.72	0.37
Oxytocin (vs. Placebo)	−0.43	(0.36)	−0.84	(0.50)	−0.61	(0.51)	−0.38	(0.46)	−0.40	0.48
ELA History	0.30	(0.45)	0.10	(0.57)	0.28	(0.69)	0.96	(0.66)	0.73	0.56
ELA History × Oxytocin	1.08^*^	(0.51)	1.71^*^	(0.76)	1.48^*^	(0.76)	1.41^*^	(0.69)	0.57	0.74
**SIMPLE SLOPES FOR SIGNIFICANT INTERACTIONS**										
Effect of OXT in ELA	0.62	(0.47)	0.86	(0.76)	0.86	(0.79)	1.15	(0.84)		
Effect of OXT in Non-ELA	−0.43	(0.24)	−0.91	(0.35)	−0.61	(0.35)	−0.38	(0.21)		

Gamma estimates from multilevel models are analogous to unstandardized beta coefficients in OLS regression. ELA, Early Life Abuse.

* p < 0.05. Simple slope significance tests are underpowered due to small subsamples; therefore, simple slopes are descriptive only.

*The bold is to emphasize statistical significance*.

To provide a metric of the size of these effects, we reran simple slope models for significant interactions using person-standardized outcomes [estimated using person-specific means and standard deviations, creating a metric analogous to a within-person *d*; (39)]. The sizes of the OXT (vs. placebo) *difference* in symptoms among women without ELA were: 0.53 standard deviation (SD) reduction in depression, 0.86 SD reduction in anger/conflict, 0.50 SD reduction in anxiety, and 0.32 SD reduction in rejection sensitivity. The sizes of the *difference* in symptoms among women with ELA were 0.75 SD increase in depression, 0.82 SD increase in anger/conflict, 0.71 SD increase in anxiety, and a 1.01 SD increase in rejection sensitivity. Given that a 1 person-SD increase is sometimes used as metric for clinically significant premenstrual symptom change, these effects would appear to suggest clinical significance. Translation of this standard-deviation based metric (within-person *d*) to *f* suggests that these interactive effects were equivalent to *f-*values ranging from 0.16 to 0.50, indicating conventionally medium-to-large effect sizes.

## Discussion

We found that early life abuse (ELA) moderated the impact of intranasal oxytocin (OXT) on daily emotional symptoms in a sample of women with symptoms of PMDD. Consistent with our hypotheses, intranasal OXT (vs. placebo) decreased PMDD symptoms in women without ELA, and increased PMDD symptoms in women with ELA.

To our knowledge, this is the first experimental intranasal OXT study to include daily symptom reports to prospectively predict specific patterns of OXT response based on history of ELA. Our results mirror previous studies showing intranasal OXT may not elicit prosocial responses in individuals with early adverse caregiving experiences (16–19), but extend these findings to a female-only clinical sample and includes prediction of daily symptom data during (premenstrual) OXT administration.

In this experiment, intranasal OXT had pronounced effects on social symptoms, including the anger/conflict composite and rejection sensitivity. Some evidence points to persistent ELA-related disruptions of stress-related/neuroendocrine systems specifically in PMDD (27, 28, 30, 31) and such abnormalities may underlie the differential effects of OXT on social-emotional cognition and behavior (11). For women without ELA, OXT may normalize dysregulation of stress circuitry and/or affiliative responses [e.g., as in Social Anxiety Disorder; (14)], thereby reducing symptoms. For women with ELA, OXT may disrupt stress regulatory capacity and increase salience to social cues, which may prime threat detection and bias, and increase risk for the interpersonal symptoms that are commonly observed in affective disorders such as PMDD (6). Of note, PMDD was chosen as a model sample due to high rates of ELA and interpersonal impairment in this population; however, we do not have reason to suspect that the effects of ELA on OXT response are somehow unique to women with symptoms of PMDD.

One prior cross-sectional study has examined how early life sexual abuse in the context of PMDD (or subthreshold PMDD) moderates the association between a single premenstrual OXT measurement (via plasma) and average premenstrual symptom severity (40). The authors found that, among women with early life sexual abuse, these “tonic” premenstrual OXT levels were higher than those without early sexual abuse. Further, they found that higher tonic levels of OXT predicted a *lower* average severity of symptoms among those with early sexual abuse only. At face value this may appear inconsistent with our finding that OXT administration (vs. placebo) increased symptoms in those with ELA; however, the previous study adopted a traitlike view of premenstrual OXT (in which tonic levels were associated with symptoms), whereas our study focuses entirely on the within-person, phasic effects of placebo-controlled OXT administration. It is very possible that the tonic and phasic effects of OXT differ among those with ELA. Future work should consider this possibility, modeling both the between- and within-person effects of OXT in longitudinal and experimental studies.

Our study should be interpreted with respect to its unique strengths and weaknesses. First, our findings should be replicated in larger samples. Notably, our study was prematurely discontinued due to a facility upgrade. Despite our small sample size, the repeated measures design allowed for 80% power to test the hypothesis that ELA and OXT exert conventionally medium (*f* = 0.25) effects on daily premenstrual symptoms. Further, effect sizes for OXT-related symptom change were suggestive of medium-to-large, meaningful therapeutic effects in women without ELA and clinically-significant worsening in women with ELA. Nonetheless, these results should be interpreted with caution until they can be replicated in a larger sample. We recruited a carefully-selected clinical sample using 2 months of daily ratings to diagnose PMDD (or subthreshold PMDD) and a highly specific interview assessing abuse experiences. Although this sample is heterogeneous with respect to comorbidities, histories of abuse, and other variables, we did not observe ELA group differences in any demographic variable, and the observed heterogeneity is the norm in psychiatric and PMDD samples. Future studies should examine the unique effects of different types of abuse (e.g., emotional, physical, sexual). While the sample was primarily comprised of women meeting prospective criteria for DSM-5 PMDD, and the premenstrual mean severity was consistent with previous large PMDD samples [e.g., (41)], one individual in each ELA group demonstrated a PMDD that did not consistently show premenstrual elevation of five total symptoms (i.e., as the DSM-5 requires). Future studies should examine effects of OXT on a broader variety of PMDD symptoms. Additionally, future studies should consider including measures of biological markers, such as resting-state or task-based fMRI, to investigate neurophysiological mechanisms that are related to specific affective or social-cognitive processes following intranasal OXT administration. Such work may elucidate biological targets for evaluating novel therapeutics as a function of ELA (42). Perhaps the most notable strength of our study is the crossover experimental design, which allows for each woman to serve as her own control in a within-person test of OXT effects on symptoms. Now, larger randomized controlled trials with double-blind procedures are needed.

This study adds to an emerging literature highlighting the role of contextual and historical factors (especially those related to stress exposure) in shaping the effects of intranasal OXT on emotion and behavior in clinical samples. Although larger trials are warranted to examine the therapeutic value of intranasal OXT in PMDD and other affective disorders, the growing number of studies highlighting the potentially adverse effects of OXT in people with stress-related clinical history markers should inform the design of such studies to avoid harm and increase benefit to participants.

## Ethics Statement

This research was conducted in compliance with the ethical standards of the American Psychological Association and with the approval of the University of North Carolina at Chapel Hill Institutional Review Board. Participants provided written informed consent before data collection.

## Author Contributions

EW developed hypotheses, conducted statistical analyses, and wrote the manuscript. TE-M conducted statistical analyses and wrote the manuscript. CP, DR, SG, and GD designed and implemented the study, and contributed to manuscript writing and editing.

### Conflict of Interest Statement

The authors declare that the research was conducted in the absence of any commercial or financial relationships that could be construed as a potential conflict of interest.
