# Supplementary material for: Early Life Abuse Moderates the Effects of Intranasal Oxytocin on Symptoms of Premenstrual Dysphoric Disorder: Preliminary Evidence From a Placebo-Controlled Trial
Source: Front Psychiatry. 2018 Nov 29;9:547. doi: 10.3389/fpsyt.2018.00547 (PMC6282546; doi:10.3389/fpsyt.2018.00547)

**Appendix.**

**Salivary oxytocin (OXT) on study visits:**

For each participant, salivary OXT was collected (via passive drool) 30min after intranasal OXT or Placebo administration. The below figure shows salivary OXT concentration for each participant on a given study visit. For 8/10 participants, salivary OXT significantly increased on OXT (vs. Placebo) administration visits, suggesting that the experiment manipulation was successful (*p* < .0001). One participant (#6) did not provide an adequate amount of saliva to determine salivary OXT concentration. For one participant (#5), salivary OXT showed negligible differences between OXT and Placebo scan visits. We reran statistical analyses described in the manuscript with participant #5 omitted; there were no substantive changes in our results when this person was removed from analyses.


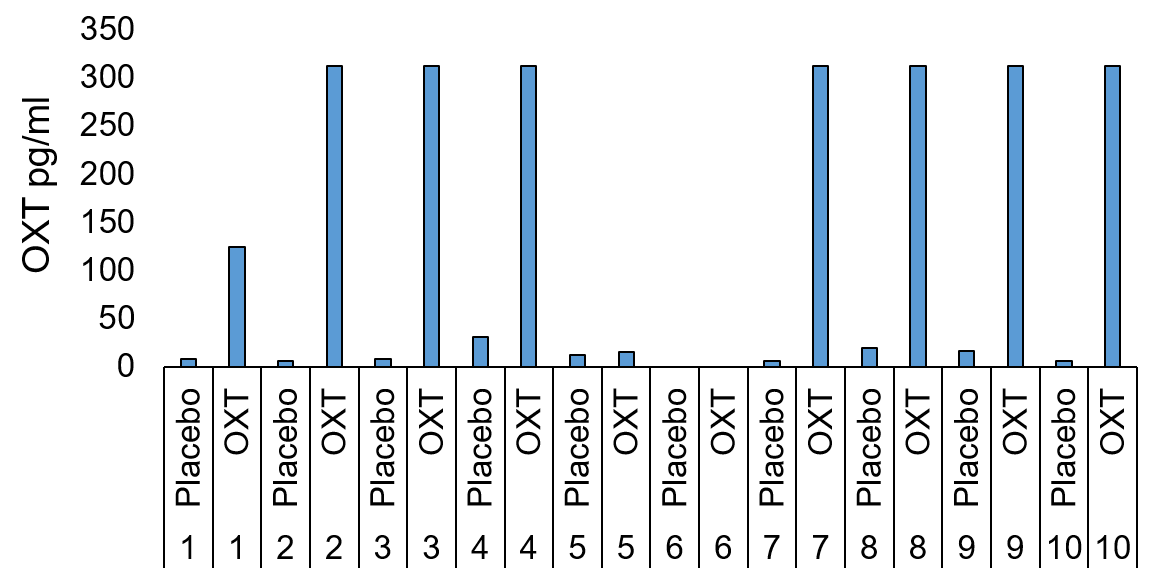

Supplement: Supplementary file 1 [file Data_Sheet_1.docx]
